# Deep learning-based framework for real-time upper limb motion intention classification using combined bio-signals

**DOI:** 10.3389/fnbot.2023.1174613

**Published:** 2023-07-27

**Authors:** A. Usama Syed, Neelum Y. Sattar, Ismaila Ganiyu, Chintakindi Sanjay, Soliman Alkhatib, Bashir Salah

**Affiliations:** ^1^Department of Industrial Engineering, University of Trento, Trento, Italy; ^2^Department of Mechatronics and Biomedical Engineering, Air University, Islamabad, Pakistan; ^3^Industrial Engineering Department, College of Engineering, King Saud University, Riyadh, Saudi Arabia; ^4^Engineering Mathematics and Physics Department, Faculty of Engineering and Technology, Future University in Egypt, New Cairo, Egypt

**Keywords:** assistive robotics, disability, intelligent systems, machine learning, prosthesis, trans-humeral amputation, sEMG and fNIRS

## Abstract

This research study proposes a unique framework that takes input from a surface electromyogram (sEMG) and functional near-infrared spectroscopy (fNIRS) bio-signals. These signals are trained using convolutional neural networks (CNN). The framework entails a real-time neuro-machine interface to decode the human intention of upper limb motions. The bio-signals from the two modalities are recorded for eight movements simultaneously for prosthetic arm functions focusing on trans-humeral amputees. The fNIRS signals are acquired from the human motor cortex, while sEMG is recorded from the human bicep muscles. The selected classification and command generation features are the peak, minimum, and mean ΔHbO and ΔHbR values within a 2-s moving window. In the case of sEMG, wavelength, peak, and mean were extracted with a 150-ms moving window. It was found that this scheme generates eight motions with an enhanced average accuracy of 94.5%. The obtained results validate the adopted research methodology and potential for future real-time neural-machine interfaces to control prosthetic arms.

## 1. Introduction

Human motion identification by monitoring muscle activation is a thriving field of research. There are various methods for tracking muscle activity that occurs during physical movement (Visconti et al., 2018). For instance, to monitor muscular contraction, techniques applied include sonomyography (SMG) (Xie et al., 2014), mechanomyography (MMG) (Sattar et al., 2021a), miokinemetric (MK) (Sattar et al., 2021a), and electric impedance estimation (Xie et al., 2014). Muscle contraction for intention is often determined using surface electromyography (sEMG) and near-infrared spectroscopy (NIRS) (Zhou et al., 2007; Herold et al., 2018), as it allows continuous muscle motion monitoring during motor actions and activities for rehabilitation. Surface electromyography (sEMG) measures the activity of motor units (MUs) or electrodes. These electrodes are placed over the skin covering the muscles. The signals extracted by these devices are influenced by many factors, including the unit of the contraction, the number and type of fibers, and the position of the motor unit action potential (MUAP) from the signal recognition (Tan et al., 2023).

Nevertheless, these characteristics are only a related subcategory of the factors that instigate inconsistencies in interpreting the time and occurrence-based statistical features of the sEMG signals. The evaluation of these factors was conducted by researchers who have obtained significant results from their analysis (Jarrasse et al., 2017). Techniques for monitoring muscle activity are employed to measure and analyze the performance of machines and equipment, as well as to evaluate the physical condition of their operators (Hyodo et al., 2012; Hong et al., 2018). Another study showed that the hybrid scheme could estimate shoulder and elbow motion with >90% accuracy and wrist, grip, and finger motion with 65%−70% accuracy (Bakshi et al., 2018). The commands were generated independently from the forearm. These techniques can be used in various industries, including manufacturing, construction, transportation, and healthcare.

The advancement of optical brain imaging has paved the way for innovative human-machine interface (HMI) techniques (Ayaz et al., 2022). It is accomplished using functional near-infrared spectroscopy (fNIRS) (Ayaz et al., 2022). fNIRS is a brain imaging technique that is non-invasive and measures alterations in oxygenated and deoxygenated hemoglobin concentrations in the brain. It uses near-infrared light to detect changes in blood flow in the brain, which can be used to study brain function. The application of this technology to robotic systems has proven promising and attracted increased attention in the medical field (Syed et al., 2021). This technique provides a way to monitor brain activity that is both indirect and non-invasive. Its portable acquisition system supports performing experiments in any environment. The fNIRS measures near-infrared light attenuation and quantifies the concentration of chromophore attained through time-based changes. It exploits the optical window in which the fundamental elements of the human body cause no major hindrance to infrared light (700–900 nm).

Moreover, it is worth noting that the process of brain activity can also be elucidated by the absorption of light by oxygenated hemoglobin (Hb) and deoxygenated hemoglobin (deoxy-Hb). These two substances play a vital role in this process and their interaction with light is crucial in understanding the functioning of the brain. The uniqueness of absorption bands of deoxygenated and oxygenated hemoglobin allows the evaluation of relative changes in hemoglobin concentration. The light attenuation is computed at a few arbitrary wavelengths (Abitan et al., 2008). The multi-wavelengths are employed due to undifferentiated absorbing coefficients at 810 nm.

In this activity, an improved Beer-Lambert law is applied to estimate the relative concentration for the entire length covered by light photons (Pancholi and Joshi, 2020). The measurement system includes an emitter for an incident beam of light and a detector to identify the reflected light. The distance between the emitter and detector positions is estimated precisely, and the brain hemodynamic condition is apprehended. The light absorption is converted into a hemodynamic response using the Beer–Lambert Law. Four steps are taken to classify brain signals and provide neurofeedback: preprocessing, feature extraction, classification, and command production. The classifier is trained using characteristics extracted from brain signals. It can be created using machine learning or deep learning methods such as artificial neural networks, convolutional neural networks, deep belief networks, long short-term memory, or a cascade of CNN and LSTM (Luo et al., 2021). While deep learning algorithms have improved performance in solving complex classification problems in brain-computer interface research, they also present a unique challenge when dealing with large datasets.

Despite remarkable development (Pfeifer et al., 2018; Pancholi and Joshi, 2019), the classic brain signal system still confronts substantial hurdles. For starters, brain signals are easily contaminated by biological (e.g., eye blinks, muscle artifacts, weariness, and attention level) and environmental (e.g., sound) artifacts (Abitan et al., 2008). As a result, extracting helpful information from distorted brain signals and constructing a resilient system that operates in various conditions is critical. Second, it must contend with the poor SNR of non-stationary electrophysiological brain signals. Due to their time complexity and the possibility of information loss, typical preprocessing or feature extraction algorithms cannot simply address low SNR (Khan and Hong, 2017; Luo et al., 2021). For example, the state-of-the-art classification accuracy (CA) for multi-class motor imagery EEG is often less than 80%. Analyzing the studies comprised of fNIRS, this value drops to 70% (Luo et al., 2021). Recently, EEG and fNIRS have been hybridized, and promising results were obtained to investigate acupuncture therapy's effects on mild cognitive impairment patients. Unique learning approaches are required to cope with dynamic data streams in brain signal systems.

The application of advanced deep learning techniques in the analysis of brain signals has yielded impressive results, demonstrating substantial progress in overcoming the challenges posed by big data analysis and the inherently erratic nature of such signals (Luo et al., 2021). Deep machine learning offers two benefits: One, it operates on raw brain inputs, bypassing time-consuming preprocessing and feature extraction. Second, using deep structures, deep neural networks may capture both representative high-level characteristics and hidden relationships. CNNs are one of the most common deep-learning models for exploring spatial information. Because of its conspicuous qualities, such as regularized structure, high spatial locality, and translation invariance, CNN is commonly utilized to uncover latent spatial information in applications like image recognition, ubiquity, and object search. CNN is intended to capture the differential relationships among the patterns associated with various brain signals. Recently, research has been dedicated to using hybrid fNIRS-sEMG systems for multiple applications, including brain-computer interfaces, sports science, and rehabilitation.

Combined fNIRS-EMG systems can provide a comprehensive picture of brain-muscle activity during movement tasks. The combination of fNIRS and EMG can improve the accuracy and reliability of brain-computer interface systems. This system can study the relationship between brain activity and muscle activity during different types of movement. Using hybrid fNIRS-EMG systems in sports science can help athletes and coaches optimize training and performance. These systems monitor brain and muscle activity during rehabilitation to maximize rehabilitation. Overall, the limited but promising literature on hybrid fNIRS-EMG systems suggests that these systems have much potential for a wide range of applications, and there is ongoing research that aims to further understand and develop these systems.

In this study, we propose to combine the information acquired from the sEMG and fNIRS to generate control commands for a prosthetic arm device designed for transhumeral amputees. These control commands are further translated to actuate the device in real-time to perform eight movements associated with the upper limb.

The structure of this paper is as follows: the contributions are outlined in the introduction, while Section 2 focuses on the acquisition and processing of experimental data for both modalities. Section 3 introduces our proposed deep-learning algorithm for recognizing human arm motions. The results are discussed in Section 4, followed by the research conclusion.

## 2. Materials and methods

### 2.1. Subjects

Twenty healthy subjects (15 males and five females) participated in the experiment (age: 32 ± 5.67). Four trans-humeral amputees also participated in the study. It was ensured that none of the subjects suffered from any past disorders concerning mental, neurological, or visual health. All subjects were briefed about the data acquisition procedure, and a consent was obtained in the written form. The details about these amputees are tabulated in Table 1. The Air University Human Research Ethics Committee (HREC) permitted trials on human subjects.

**Table 1:** Demographic details of the amputated subjects.

**Subject ID**	**A1**	**A2**	**A3**	**A4**
Gender	Male	Male	Male	Male
Age	23	27	32	45
Amputated side	Right	Left	Right	Right
Residual length	15 cm	17 cm	10 cm	18 cm
Cause of amputation	Accident	Accident	Accident	Diabetes

**Figure 1:**
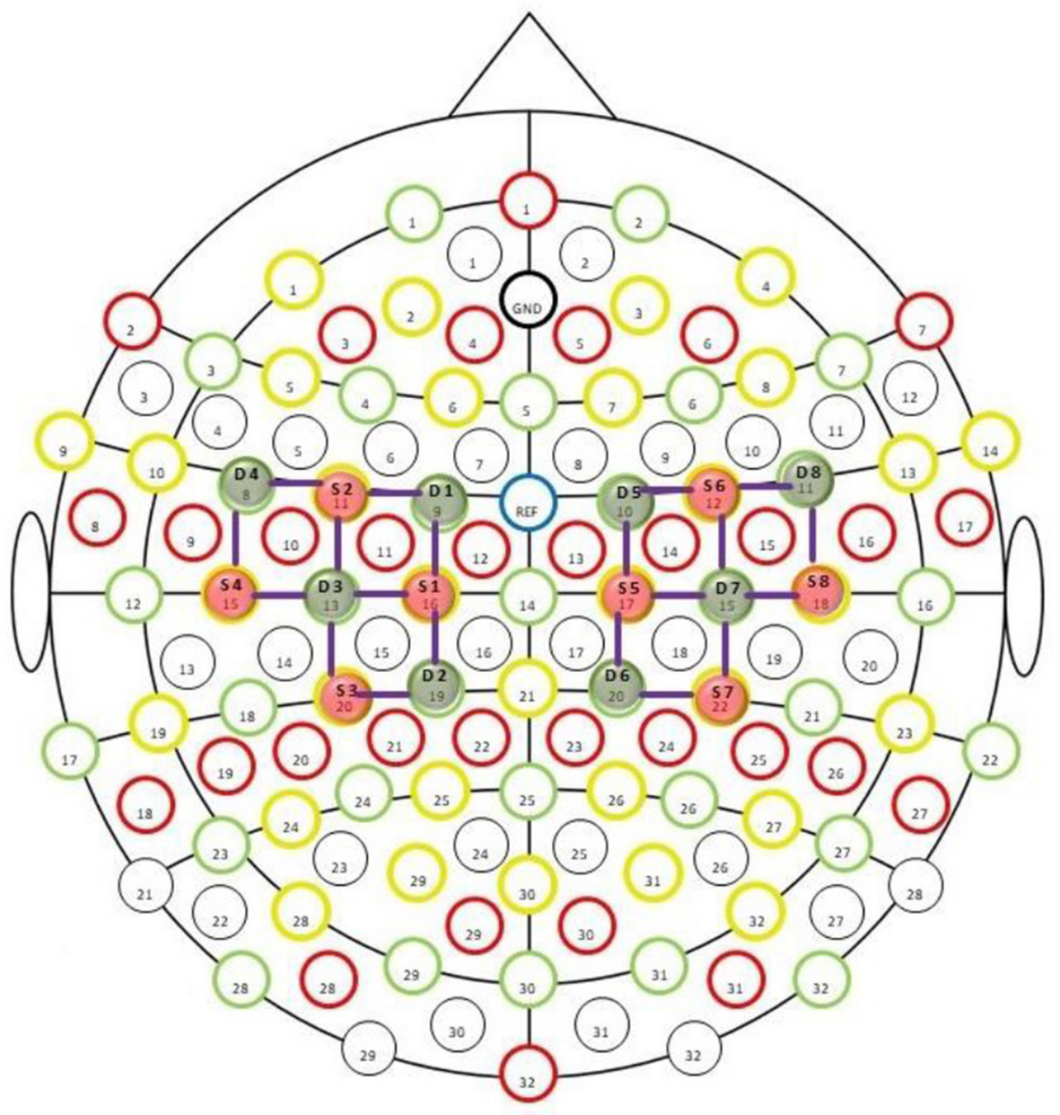
fNIRS sensors placement montage provided by NIRx technologies. The green circles labeled as D_n_ (where *n* = 1–8) are detectors, while the red circles marked as S_n_ are sources. The purple lines show the channels of interest.

**Figure 2:**
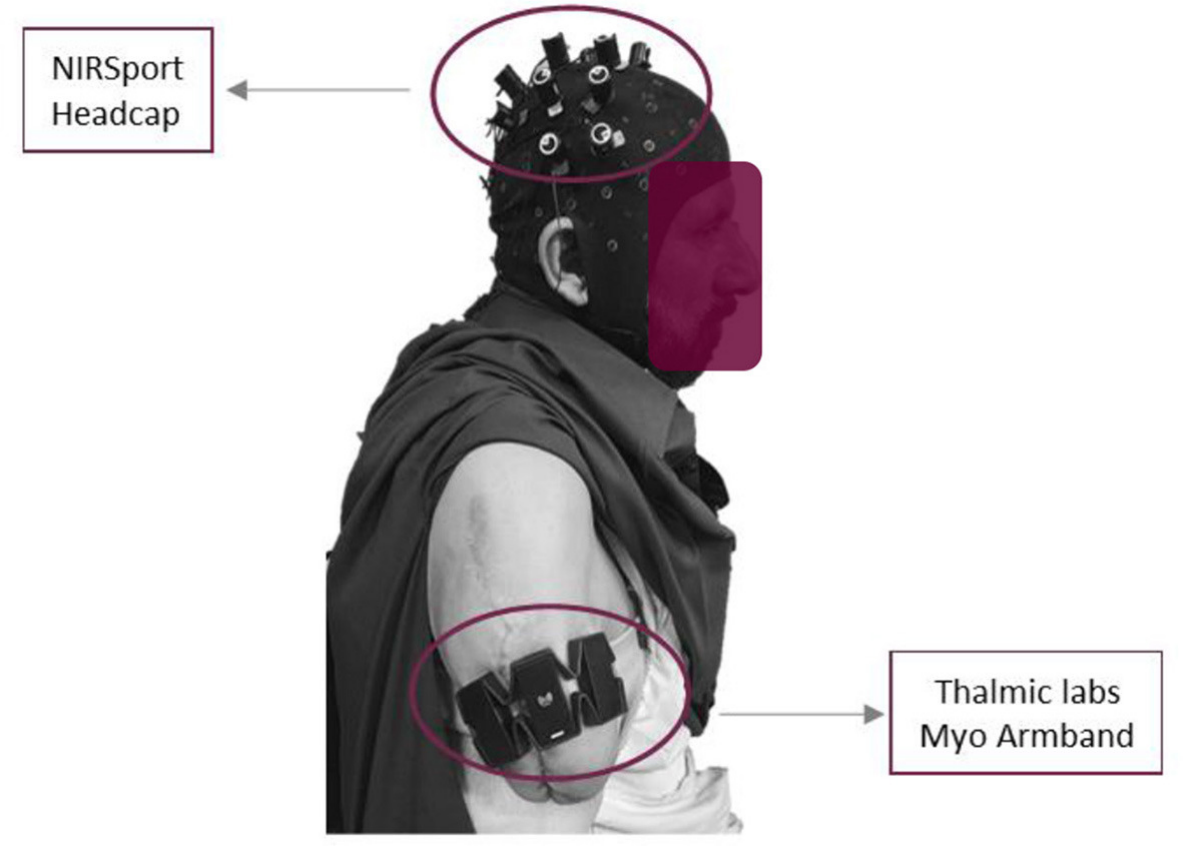
Sensors placement on trans-humeral amputated subject.

Further, these research experiments followed the ethical standards of the Declaration of Helsinki. The sensors' placement on the scalp is illustrated in Figure 1. The placement of an armband for sEMG and a head cap for fNIRS bio-signals on a transhumeral amputee subject are shown in Figure 2.

### 2.2. Sensors description

Myo armband is a wearable gadget produced by Thalamic Labs. It consists of eight sEMG sensors. The armband can receive muscle intention data at precisely the sampling frequency of 200 Hz. The Myo armband comprises a nine-axis inertial measurement unit (IMU) which contains a gyroscope and an accelerometer. All these devices possess three-axis motion and help locate the armband and acceleration position for a particular time window. It records the myoelectric intentions in milli volts (mV) from −30 mV to +70 mV. The armband was worn around the bicep muscle just above the elbow joint. However, amputee subjects used the residual muscle to wear the armband.

The NIRSport (NIRSport, NIRx Medical Technologies, LLC, United States) is a neuroimaging device with 24 detectors and 24-source optodes. We used the fNIRS optodes location decider (fOLD) toolbox to design probe arrangements covering the motor cortex. After setting the specificity threshold to 15%, we obtained eight sources and eight detectors assigned according to the 10–5 international system. This setup provided 20 fNIRS channels for measurement at the inter-optode distance of 3 cm and allowed the acquisition rate of 7.8 Hz. An easy cap with fNIRS optodes inserted at the 10–5 international system was placed between nasion to inion and left to right preauricular points (reference point Cz).

### 2.3. Experimental procedure

To account for trans-humeral amputation, four of the associated main arm motions, i.e., elbow, two wrist joint motions, and the hand motion, are considered in bi-direction. These motions compromise Wrist Extension (WE), Elbow Flexion (EF), Wrist Supination (WS), Wrist Flexion (WF), Elbow Extension (EE), Wrist Pronation (WP), Hand Open (HO), and Hand Close (HC).

### 2.4. Training session

**Figure 3:**
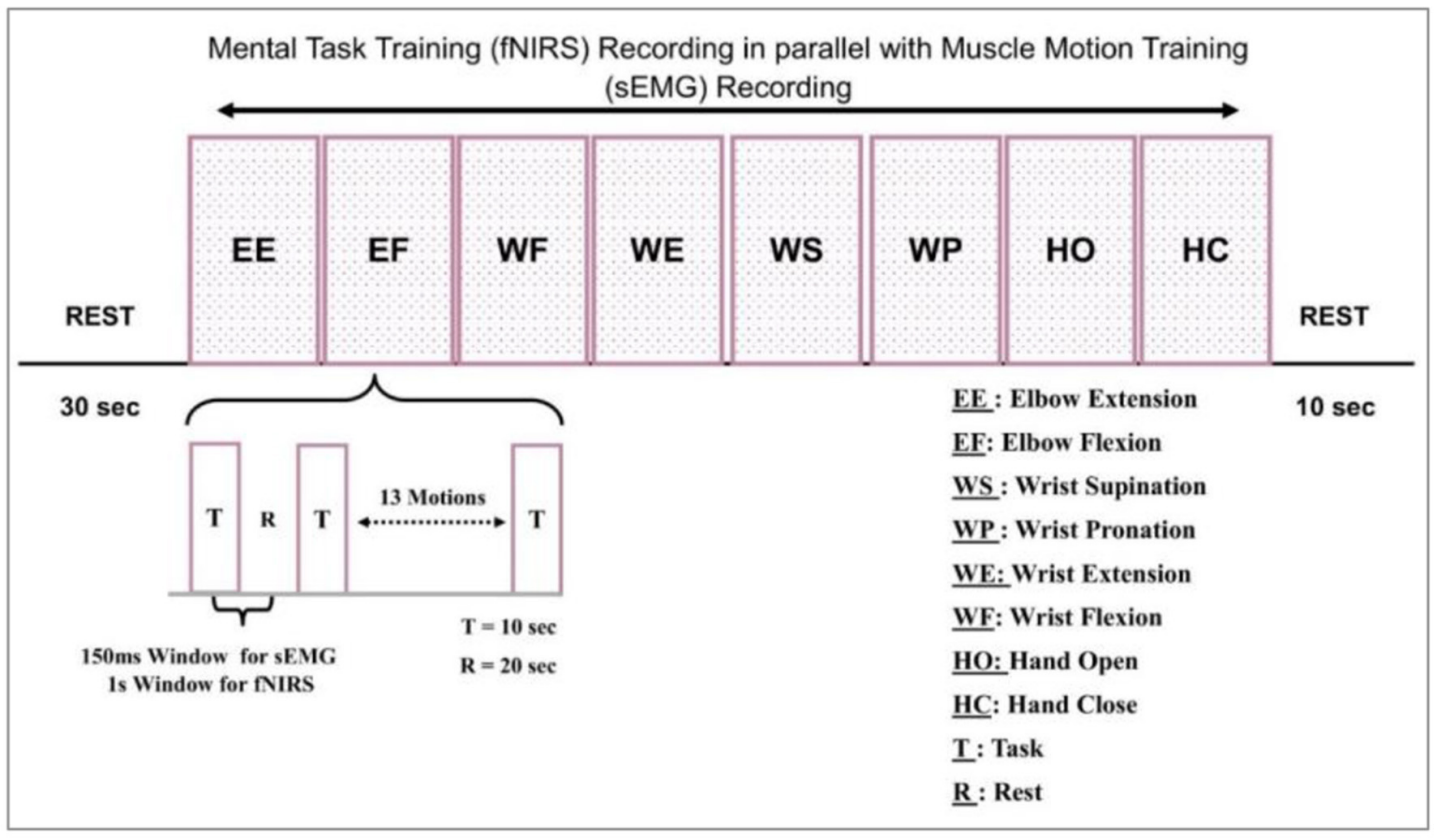
Experimental model for signal acquisition.

Before the experiment, all participants were informed of the details of experimental tasks and procedures. In addition, there was a 10 min pre-training to familiarize them with operation and training modes. The testing session included positioning the subjects on a comfortable chair about 100 cm from the table. This distance was set to avoid interference between the screen backlight and optical sensors. Another reason was to clear the visibility of motion cues prepared to assist the participant. This environment supported the signal extraction process. The data was collected in a controlled environment. The signal acquisition took place in a dark room, with the specially designed head cap to cover the optodes as the light from the laptop might interfere. The gain values computed by NIRSport were also analyzed. The data from each subject, i.e., healthy and amputee, was acquired thrice. The sampling paradigm was comprised of five tasks, including (1) an undeveloped session spanned over 30 sec to create a reference point, (2) a Screen indication to the participants to perform one of eight definite tasks, (3) recording the pre-defined tasks sequentially and then repeating the same by the subjects with random intentions, (4) recording all the eight motion's data by fNIRS and sEMG simultaneously, and (5) separating each 10-s task by a 20-s rest session. This experimental model is represented graphically in Figure 3.

The motions were repeated twice, i.e., one activity was performed 16 times by each participant. Every block in Figure 3 represents the acquisition of these motions. One block represents 16 repetitions of one movement. The sub-block representation is given in Figure 3 as well.

### 2.5. Signal acquisition and processing

Data collection: fNIRS data is collected using a system that includes light emitters and detectors. The light emitters send near-infrared light through the scalp and skull, and the sensors measure the amount of light absorbed by the brain. Data preprocessing: The raw data collected by the fNIRS system must be preprocessed to remove artifacts and noise. It includes detrending, baseline correction, and spatial filtering. Once the data has been preprocessed, it can be analyzed using various techniques. These may include statistical tests to identify changes in brain activity or more complex approaches such as machine learning algorithms. Subsequently, in the nirslab environment, the data is processed with a built-in signal processing software with NIRSport. The nirslab environment undertakes the specifications and conditions adopted in real-time and hence becomes an optimal choice for signal processing through NIRSport. The unwanted data and unusual spikes are truncated and filtered to compute the hemodynamic states.

**Figure 4:**
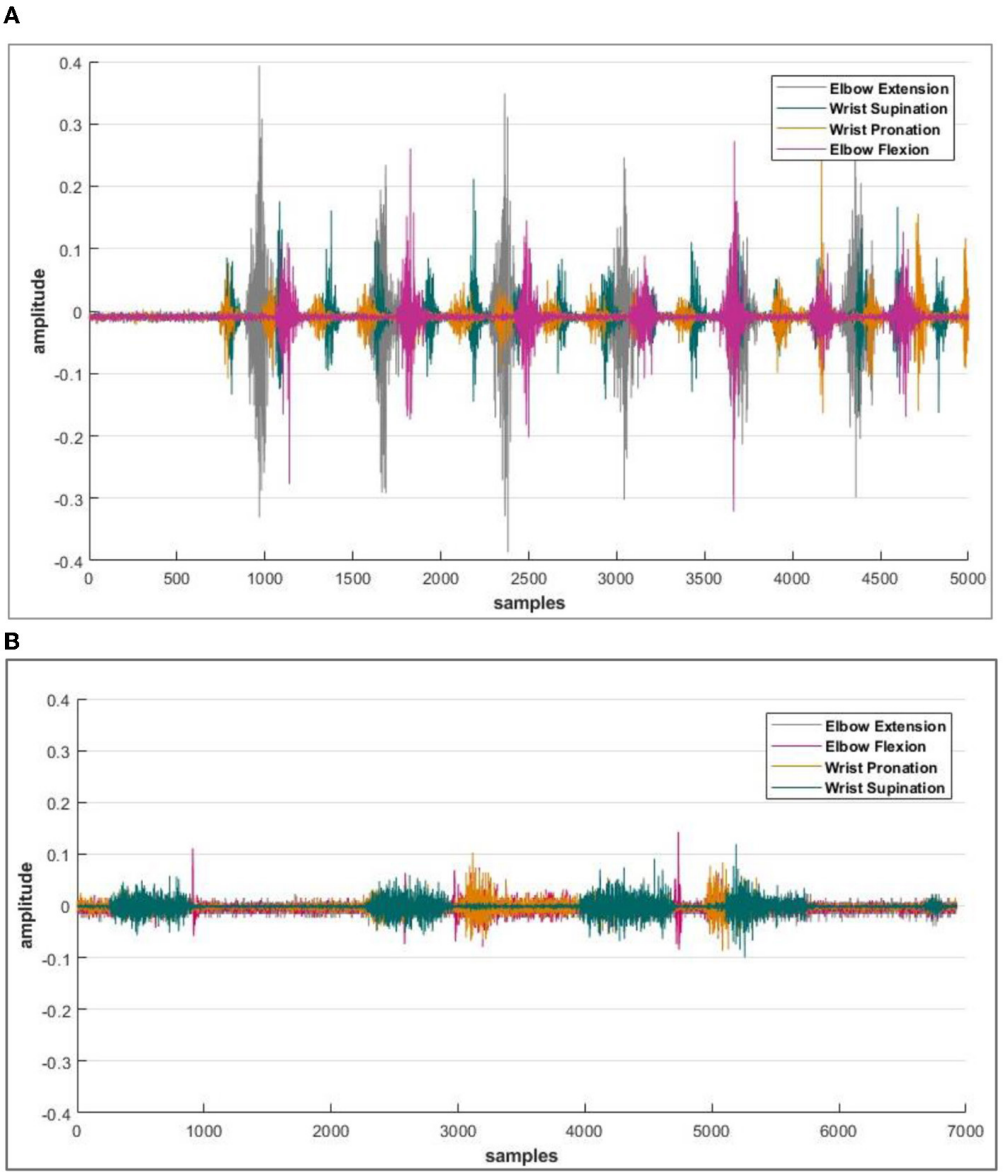
**(A)** Acquired sEMG bio-signals from healthy subjects against each defined motion. **(B)** Acquired sEMG bio-signals from an amputee subject. The amplitude of intentions acquired from the sensor is recorded in milli volts (mV), and the data is then normalized. Whereas on the *x*-axis, the number of samples is plotted.

When data logging was initiated, the next step was to find the electrodes participating in the signal acquisition process. A specific muscle was activated for one particular activity; hence, the electrode selection step reduced the time response for further actions. Reducing the amount of information transmitted by the controller results in a faster response time for signal processing. The electrode-wise activity of the Myo armband or the electromyographic intentions of a healthy and amputee subject is shown in Figures 4A, B, respectively.

It was observed that during the signal acquisition process, not all the electrodes were activated. A unique set of electrodes showed activity with each selected motion. The threshold value was set to 48 mV (by *z*-score method) to select only those channels from which important data of electromyographic signals could be recorded for healthy subjects. Meanwhile, this threshold value varies for amputees and reduces to 16 mV. It is important to take note that both the healthy and amputated subjects participated in selecting the same electrodes for identical motion. This electrode selection contributed to the generation of results with less response time. An indication of activated electrodes during all eight movements acquired from a healthy subject is illustrated in Table 2, and amputee subjects are given in Table 3.

**Table 2:** Activated electrodes of Myo armband for the selected motions of EE, EF, WS, WP, WE, WF, HO, and HC using the *z*-score method.

**Electrodes**	**EE**	**EF**	**WS**	**WP**	**WE**	**WF**	**HO**	**HC**
1	**68 mV**	40 mV	48 mV	**55 mV**	9 mV	6 mV	7 mV	10 mV
2	**72 mV**	32 mV	**54 mV**	19 mV	48 mV	7 mV	4 mV	7 mV
3	31 mV	**78 mV**	**70 mV**	20 mV	8 mV	**50 mV**	11 mV	5 mV
4	**50 mV**	19 mV	21 mV	24 mV	22 mV	30 mV	10 mV	8 mV
5	**51 mV**	17 mV	15 mV	**56 mV**	33 mV	29 mV	10 mV	13 mV
6	28 mV	**75 mV**	**52 mV**	11 mV	**49 mV**	**48 mV**	12 mV	3 mV
7	**60 mV**	**66 mV**	38 mV	**52 mV**	10 mV	15 mV	5 mV	4 mV
8	45 mV	**75 mV**	**49 mV**	31 mV	13 mV	14 mV	8 mV	10 mV

Selected/significant channels are represented in bold.

**Table 3:** Activated electrodes of Myo armband for the selected motions of EE, EF, WS, WP, WE, WF, HO, and HC using the *z*-score method.

**Electrodes**	**EE**	**EF**	**WS**	**WP**	**WE**	**WF**	**HO**	**HC**
1	20 mV	19 mV	27 mV	7 mV	−12 mV	−15 mV	–	–
2	24 mV	11 mV	6 mV	−2 mV	27 mV	−14 mV	–	–
3	10 mV	30 mV	22 mV	−1 mV	−13 mV	29 mV	–	–
4	29 mV	−2 mV	0 mV	3 mV	1 mV	9 mV	–	–
5	3 mV	−4 mV	−6 mV	8 mV	12 mV	8 mV	–	–
6	7 mV	27 mV	4 mV	−10 mV	28 mV	27 mV	–	–
7	12 mV	18 mV	17 mV	4 mV	−11 mV	−6 mV	–	–
8	24 mV	27 mV	28 mV	10 mV	−8 mV	−7 mV	–	–

When moving from the elbow to the wrist, fewer electrodes are activated because the bicep muscles struggle to accurately capture wrist movements. Therefore, it is crucial to extract pertinent data features from the signals that have been recorded. In case of weak electromyographic intention, the wavelength feature is prudent to generate accurate results not commonly obtained by other attributes, i.e., mean, peak, etc.

In EMG signal processing, it is common to use a sliding window approach in which a window of a certain length is moved along the length of the signal, and various calculations are performed on the data within the window at each position. The window size can significantly impact the results of these calculations, and no “correct” window size is appropriate for all applications. Some standard window sizes that are used for EMG signal processing include (Barron et al., 2020):

- 50–200 ms: These window sizes are often used for time-frequency analysis of EMG signals, as they are small enough to capture the fine temporal structure of the signal but large enough to provide a reasonable amount of data for analysis.
- 500–1,000 ms: These window sizes are often used for calculating statistical measures of EMG signals, such as mean power, variance, and kurtosis.
- 2,000–5,000 ms: These window sizes are often used for analyzing the overall activity of an EMG signal, such as calculating the total number of muscle contractions or the entire time a muscle was active.
- Whereas some standard window sizes used for fNIRS signal processing include: 2 s, 5 s, 10 s, and 30 s.

The nirslab package is a robust MATLAB-based software analysis environment designed to aid in studying time-varying near-infrared observations. It imports NIRS measurement data and pertinent information about the measurement [e.g., positions of optical probes (optodes) on the scalp, as shown in Figure 1. time of applied stimuli or experimental tasks]. Creating files containing optode-position and measurement-timing information and toolboxes for modifying this data. Preprocessing measurement data with toolboxes that eliminate noisy data channels erasing empirically unnecessary periods, removing artifacts from data, and filtering to exclude experimentally irrelevant frequency bands.

Since limb movement is involved, motion artifacts may get involved in the acquired data. Several approaches can be used to remove motion artifacts from (fNIRS) data (Pfeifer et al., 2018).

In some cases, excluding data points heavily contaminated by motion artifacts may be necessary. It is done by visually inspecting the data or using automated algorithms to detect and flag these data points. However, the present study is based on capturing the fNIRS because of movements, so these artifacts cannot be filtered out as it may cause aninformation loss. However, the measures mentioned above can be taken to avoid such artifacts. Several steps are involved in processing fNIRS data (Cui et al., 2010; Scholkmann and Wolf, 2012). These steps are described in the coming sections.

An FIR (finite impulse response) filter is a digital filter commonly used to process signals in various applications, including audio, image, and biomedical signal processing (Syed et al., 2020). FIR filters are characterized by having a finite impulse response, i.e., the output of the filter will eventually become zero after a limited number of time steps. FIR filters are implemented using a set of coefficients to weigh the input data. These coefficients are designed to emphasize specific frequencies in the input signal and attenuate others, depending on the desired frequency response of the filter. The output of an FIR filter can be expressed as:


(1) $$\begin{array}{l} y\left[n\right]\;=\;\sum \text{\_}k\;x\left[n-k\right]{\;}^{*}h\left[k\right] \end{array}$$

where *y*[*n*] is the output at a time step *n, x*[*n*] is the input at a time step *n*, and *h*[*k*] is the *k*-th coefficient of the filter.

The choice of window function will affect the trade-off between the stopband attenuation and the transition width of the filter. Different window functions are suitable for various applications, and selecting the appropriate window function is an essential consideration in designing an FIR filter. In the presented study, the *z*-score has been evaluated for electrode/optode selection. The *z*-score method, also known as the standard score method, is a statistical technique used to determine how many standard deviations a given data point is from the mean of a dataset (Zhang et al., 2019). The *z*-score is calculated as follows:


(2) $$\begin{array}{l} z\;=\;\left(x\;-\;\mu \right)/\sigma \end{array}$$

where *x* is the data point, μ is the mean of the dataset, and σ is the standard deviation of the dataset.

The *z*-score can identify outliers in a dataset and determine whether a data point is statistically significant. For example, a data point with a *z*-score of 2 or more is an outlier because it is more than two standard deviations from the mean. Similarly, a data point with a *z*-score of 1.96 or more in a two-tailed test is statistically significant at the 0.05 level (Khalil et al., 2022). Then the features are extracted from the data samples and used as input to neural networks for motion prediction training (Zhang et al., 2021).

Sub-HbOdynamic changes are plotted in real-time on NIRStar^®^ and computed offline in nirslab. The results were based on the modified Beer-Lambert law for scattering media. The operator modified all input parameters of the Beer-Lambert law (absorption coefficients and inter-optode distance) in nirslab. In NIRStar^®^, these input parameters were fixed according to the values calculated in real-time. The default inter-optode distance was set to 3.0 cm. A change in the light reduction at a known wavelength is expressed by equation (3). The MBLL was used to convert changes in raw optical density signals into oxy- and deoxy-hemoglobin concentration changes, respectively (*c*_*HbO*_(*t*) *and c*_*HbR*_(*t*)):


(3) $$\begin{array}{l} \left[\frac{\Delta c_{HbO}\left(t\right)}{\Delta c_{HbR}\left(t\right)}\right]=\frac{{\left[\begin{array}{c} \alpha_{HbO}\left(\lambda_{1}\right)\&\alpha_{HbO}\left(\lambda_{1}\right)\\ \alpha_{HbO}\left(\lambda_{2}\right)\&\alpha_{HbO}\left(\lambda_{2}\right) \end{array}\right]}^{-1}\left[\begin{array}{c} \Delta A\left(t;\lambda_{1}\right)\\ \Delta A\left(t;\lambda_{2}\right) \end{array}\right]}{l\times DPF} \end{array}$$

Differential path-length factor (DPF) denotes the curve path length factor, and *l* is the distance between the source and detector. *A*(*t*; λ_1_) and *A*(*t*; λ_2_) are the absorption at two different instants, α_*HbO*_(λ) and α_*HbR*_(λ) are the extinction coefficients of HbO and HbR, while Δ*c*_*HbO*_(*t*) and Δ*c*_*HbR*_(*t*) represent changes in the concentration of HbO and HbR, respectively (Strangman et al., 2002, 2003; Ando et al., 2010; Cui et al., 2010; Scholkmann et al., 2010; Scholkmann and Wolf, 2012; Albinet et al., 2014; Lambrick et al., 2016).

**Figure 5:**
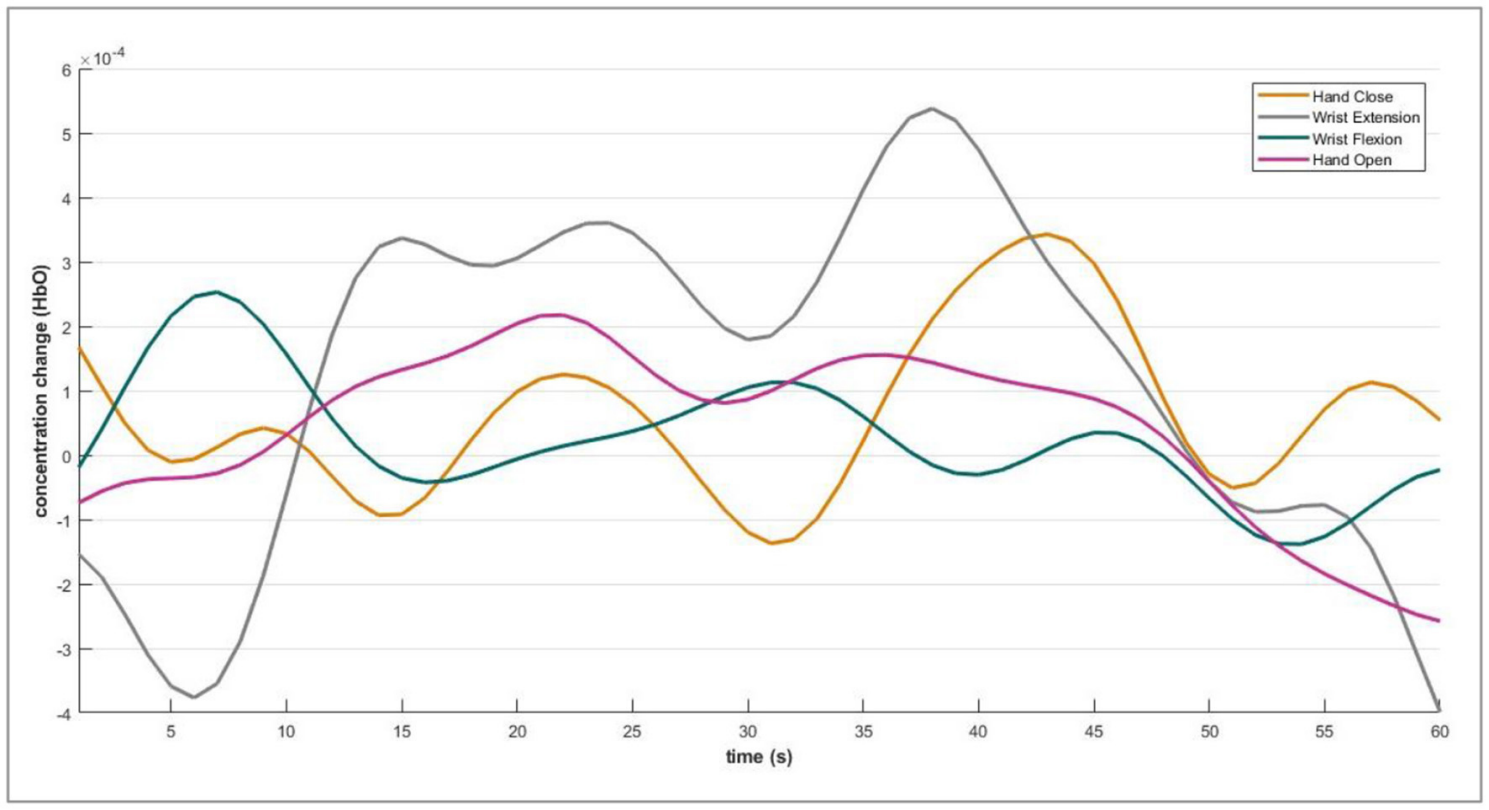
Acquired fNIRS bio-signals against each defined motion.

The DPF is a dimensionless modification factor. The DPF detects an increase in the optical path length. The optical path length is produced by light scattering in organic tissue. The DPF and source-detector separation product evaluate the “true” path length of the light inside the biological tissue cell (Villringer and Chance, 1997; Perrey, 2008; Issard and Gervain, 2018; Quaresima and Ferrari, 2019). For NIRx technologies, the value of optical path length is set constant for wavelengths. The fNIRS channel-wise hemodynamic states are demonstrated in Figure 5.

### 2.6. Deep learning scheme

This section highlights the architecture of a deep learning algorithm implemented in this research to translate the selected eight motions for the upper limb.

#### 2.6.1. Deep neural network architecture

Deep neural networks have more layers than traditional ones (Luo et al., 2021; Sattar et al., 2021a; Zhang et al., 2021). The layers in a deep neural network are composed of multiple artificial neurons, each connected to several other neurons in the next layer. In the human brain, the neurons inspire the artificial neurons in a deep neural network. They are intended to process and transfer information in a manner similar to how organic neurons function.

Convolutional neural networks (CNNs) are a type of deep neural network that process data with a grid-like structure. CNNs comprise several layers, including convolutional, pooling, and fully connected layers. A few fundamental equations that are commonly used in CNNs (Li et al., 2017; Syed et al., 2021) are given as:

- Convolution: This mathematical operation combines the input data with a set of learnable weights (kernel or filter) to produce a feature map. The convolution operation can be expressed mathematically as in equation (4):

(4) $$\begin{array}{l} F\;\left[i,\;j\right]\;=\;\sum \text{\_}k\;\sum \text{\_}l\;X\left[i+k,\;j+l\right]{\;}^{*}W\left[k,\;l\right]\;+\;b \end{array}$$

F is the feature map, *X* is the input data, *W* is the kernel, and b is a bias term.

- Pooling: This down-sampling operation reduces the feature map size and the number of parameters in the model. There are several types of pooling, including max pooling and average pooling. Max pooling can be expressed as equation (5):

(5) $$\begin{array}{l} F^{\prime }\left[i,\;j\right]\;=\;max\;\left(F\;\left[is:is+f,\;js:js+f\right]\right) \end{array}$$

where *F*′ is the pooled feature map, *s* is the stride size, and *f* is the size of the pooling window.

- Fully connected layer: This is a traditional neural network layer used to interpret the features extracted by the convolutional layers and make a prediction. The output of a fully connected layer can be expressed as equation (6):

(6) $$\begin{array}{l} F=WX+b \end{array}$$

*F* is the layer's output, *W* is the weight matrix, *X* is the input data, and *b* is a bias term. During the experimental studies, it was analyzed that raw data produced by the selected signal is ineffective for analysis because of its non-linear, non-stationary, and stochastic nature. Such characteristics of the sEMG-fNIRS signals are due to the continuous variation in motor unit recruitment and the arbitrary way the motor unit potentials are superimposed.

The two main factors that engender a change in these signals include (1) the number of peaks (NPs), which corresponds to the frequency of components, and (2) the signal's amplitude, which directly relates to the strength of a particular activity. The parameters used to derive the proposed scheme include (1) *T* acts as the window size or frame size, and (2) the nth derivative of the signal, i-e, △^*n*^, (3) the discrete Fourier transform (DFT) of signals is represented as χ[k], and (4) the frequency index is defined by *k*, where *k* varies from 0 to *T* – 1. The time differential property of the Fourier Transform (Feng et al., 2020) indicates that the nth derivative of the signal is computable and is calculated by multiplying the signal's frequency spectrum and nth power of *k* (Li et al., 2017; Ameri et al., 2019), as explained in (7).


(7) $$\begin{array}{l} F\left[{△}^{n}\sigma \left(t\right)\right]=k^{n}\chi \left[k\right] \end{array}$$

where σ(*t*) is the signal in the time domain, △^*n*^ is the nth derivative of the signal, and χ [*k*] is the frequency transform of the signal. The power spectral moments are used to preprocess the signal before feeding input to the neural network. The moment μ of the nth order is defined as (8).


(8) $$\begin{array}{l} \mu_{n}=\sqrt{\overset{T-1}{\underset{t=0}{\sum }}t^{n}\chi \left[t\right]}. \end{array}$$

According to the integral of squares method by Parseval's theorem, the segmented signal is defined in the form of its power as in (9)


(9) $$\sum_{t=0}^{T-1}\sigma [t]=\frac{1}{T}\sum_{t=0}^{T-1}|\chi [t]\cdot \chi {\left[t\right]}^{*}|=\sum_{t=0}^{T-1}\eta [t]$$

Using the equations (7), (8), and (9), the 0th, second, and fourth moments are defined as follows in (10) and (11)


(10) $$\begin{array}{l} \mu_{0}=\sqrt{\overset{T-1}{\underset{t=0}{\sum }}{\left(\sigma \left[t\right]\right)}^{2}} \end{array}$$

(11) $$\begin{array}{l} \mu_{2}=\sqrt{\overset{T-1}{\underset{t=0}{\sum }}{\left(\Delta \sigma \left[t\right]\right)}^{2}} \end{array}$$

Similarly, the fourth-order moment is expressed by equation (12).


(12) $$\begin{array}{l} \mu_{4}=\sqrt{\overset{T-1}{\underset{t=0}{\sum }}k^{4}\chi \left[t\right]}=\sqrt{\overset{T-1}{\underset{t=0}{\sum }}{\left(\Delta^{2}\sigma \left[t\right]\right)}^{2}} \end{array}$$

The number of peaks (NP) is then computed using the ratio of moments (Pancholi and Joshi, 2019). As defined in equation (13)


(13) $$\begin{array}{l} NP=\;\sqrt{\frac{\mu_{4}}{\mu_{2}\;}} \end{array}$$

The square version of NPs is shown in equation (14)


(14) $$\begin{array}{l} NP=\;\sqrt{\frac{\mu_{4}}{\mu_{2}}=\;\beta } \end{array}$$

(15) $$\begin{array}{l} PP=\;\mu_{O}^{*}\beta \end{array}$$

In the proposed architecture, the sEMG-fNIRS signal passes through a function named PP (β) or the product of peaks and power as expressed in equation (15). This preprocessing step forms the bio-signal stationery and reduces the dimension of the training dataset with less neural information loss.

**Figure 6:**
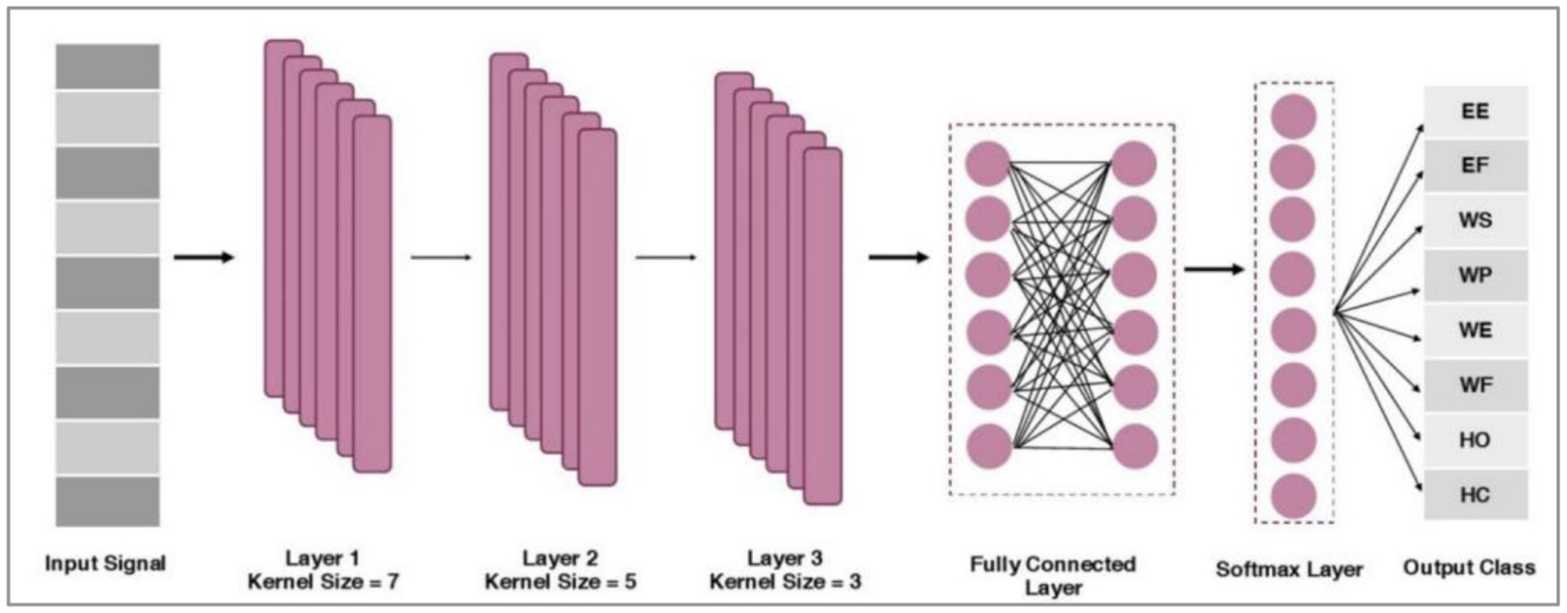
Proposed CNN architecture.

The proposed network comprises three convolution layers, two fully connected layers, and one SoftMax layer. These layers are illustrated in Figure 6. First, preprocessed information is passed through the first convolution layers that contain 128 filters with a kernel size of 7. The output from the first convolution layer constitutes 18 × 128. The second convolution layer includes the same number of filters with a kernel size of 5. The output from the second convolution layer is 14 × 128. Subsequently, the max pool of size two was incorporated into the architecture, and an output of 7 × 128 was obtained.

Further, a third convolution layer with 64 filters with a kernel size of 3 is applied. The output of the third convolution layer is 5 × 64. Afterward, the global average pooling is applied between the third and fourth fully connected (FC) layer of size 512. An FC of 128 is incorporated by following the SoftMax layer and adopting the Adam optimization method (Wen et al., 2021).

For layer 1, the mathematical formulation is described as (16).


(16) $$\begin{array}{l} \alpha_{m}^{l}\left(j\right)=b^{l}\left(j\right)+\overset{i\leq 128}{\underset{i=1}{\sum }}\left(I_{i,j}w_{1}\left(p,q,i\right)+I_{i,j+1}w_{2}\left(p,q,i\right)\right) \end{array}$$

where $\alpha_{m}^{l}\left(j\right)$ belongs to neuron j of map q in the respective layer *l*, it also shows the multiplication between the weighted values and input neurons. The filter used in the neural network is *w*_*k*_ (*p, q, i*) layer, map, channel/kernel, and the positions are defined by *p, q, i*, and *k* in the kernel, respectively. The bias in layer-*1* for neuron *j* is indicated by bl(*j*). For layer-2 and layer-3, the mathematical expression is given as equation (17).


(17) $$\begin{array}{l} \alpha_{m}^{l}\left(j\right)=b^{l}\left(j\right)+\overset{i\leq N_{k\in rnel}^{l}}{\underset{i=1}{\sum }}\left(M_{i,j}w_{l}\left(p,q,i\right)\;+M_{i,j+1}^{l-1}w_{2}\left(l,m,i\right)\right) \end{array}$$

Nlkernel denotes the number of _kernel_s in layer *l*. *M*_*i, j*_ belongs to the components *i, j* (neuron *j* in-kernel *i*) of the feature maps produced, following the convolution from the preceding layers. Finally, two fully connected layers (FCs) are used, and a SoftMax layer of size eight is employed. After this, the information acquired from the bio-signals is fed into a classifier to map distinct patterns. After classifying the data, their real-time testing was performed to validate the significance of training performance.

Following the completion of classifier training, the outputs are merged to arrive at a conclusive decision. The forthcoming section will outline the methodology employed to establish a definitive conclusion from both modalities. Section 4 will contain an in-depth analysis of the outcomes derived from this evaluation.

## 3. Combining bio-signals

A hybrid fNIRS-sEMG system combines these two techniques to measure both brain and muscle activity simultaneously. Some of the key findings from this research include that Hybrid fNIRS-sEMG systems can provide a complete picture of brain and muscle activity during movement tasks (Khan and Hong, 2017; Nsugbe et al., 2020; Sattar et al., 2021b). The combination of fNIRS and sEMG can improve the accuracy and reliability of brain-computer interface systems. Hybrid fNIRS-sEMG systems can be used to study the relationship between brain activity and muscle activity during different types of movement. Using hybrid fNIRS-sEMG systems in sports science can help athletes and coaches optimize training and performance. Combining fNIRS and sEMG technologies in hybrid systems allows for monitoring of both brain and muscle activity during rehabilitation, which can optimize the rehabilitation process. Current research on these systems shows promising potential for various applications, and continued efforts are being made to further understand and enhance them.

A fundamental impression behind this hybrid fNIRS and sEMG-based control interface is combining both modalities to mutually counter their shortcomings. Specific inadequacies have been reported in published literature when a single modality is used for bio-signals (Villringer and Chance, 1997; Nsugbe et al., 2020). A single output from two signals is achieved in a few unique manners and may include the devices' explicit applications and limitations (Park et al., 2023; Pichiorri et al., 2023). Hybrid methods are implemented to run a simple game control for a healthy person, which may aid in control applications of peripheral devices used by amputees.

Usually, the fNIRS or surface electromyographic intention signals are examined to activate specific modules of any hardware, such as elements in an assistive device (Zhang et al., 2019, 2021). On the other hand, both could be merged. The final output allows users to adjust and switch control reliably and efficiently. Only limited approaches can be applied to classify the hybrid sEMG- fNIRS intentions for a specific peripheral appliance. A hybrid scheme processes the predicted input commands simultaneously or sequentially as a multiple-input framework.

The number of classes has an impact on the accuracy of fNIRS estimation; as the number of classes rises, the accuracy declines as the brain hemodynamic responses become uniform as a signal. On the other hand, a classifier distinguishes the motion intention based on sEMG, even if the number of classes is more than 50. Hence, the control is distributed to both modalities. The hand motions could not be predicted in the present study as the signal source for the bicep muscle of trans-humeral amputee limits the number of motion intentions. As the inference is based on the learning model, the fNIRS can counter this problem by giving the motion intentions of the hand, especially in the case of amputees.

## 4. Results and discussions

The state-of-the-art research suggests that the human-machine interface controls are initiated either on a single modality or through the hybridization of multiple modalities. The present study lodges on a hybrid method to generate the control commands translated to activate a prosthetic arm for trans-humeral amputees. The obtained results in this research are briefly discussed below.

### 4.1. Channel selection

The results show that all the optodes were not apprehending the actual concentration change when the subject executed brain activity. However, the same channels were active when similar motions were performed in another trial of signal acquisition. Hence, Channel activations were sorted according to the *t*-values extracted from the training data.

It is important to note that the choice of window size can significantly impact the analysis results and should be carefully considered based on the specific goals of the study. Window sizing is generally proposed to generate a fast response for real-time applications (Khalil et al., 2022). Considering this, the 0–0.5, 0–1, and 0–2 s for windows were selected. The split-seconds were employed for sEMG features extraction and investigation of hemodynamic features to secure the best window size to decrease the computation time.

The channel outputs highlight the need to choose good channels for recognizing definite brain activitie. The Signal averaging and *z*-score method are used to select a channel as per the channel choice standard. This concept has been supported by human brain studies (Hong et al., 2018; Park, 2023; Park et al., 2023). When the right side of the human body is in motion, the human brain's left hemisphere is activated. In this research, the subjects were asked to move their right arm, and it is evident that the hemodynamic patterns occurring in the right hemisphere were merely noisy. The right hemispheres' channels (1–10) do not show significant activity. Considering this, the action was discarded in feature extraction and motion classification. However, the left side of the motor cortex was active, and the channels from 11 to 20 were used to classify the motions.

### 4.2. Classification accuracy

The performance outcome was statistically evaluated based on the number of correctly predicted samples. This activity was completed during 0–10 s, and data was gathered from sEMG and fNIRS.

**Figure 7:**
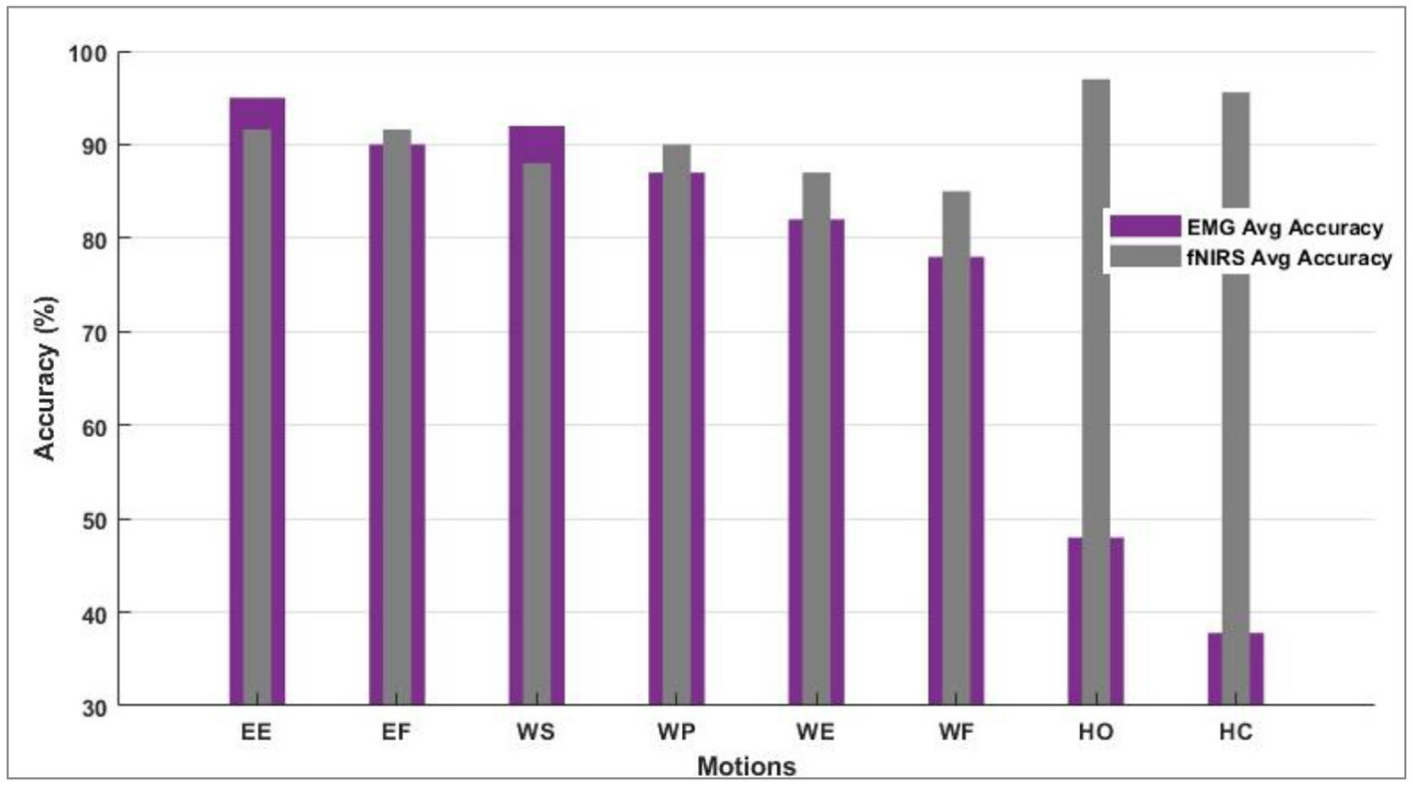
Motion-wise accuracies of individual subjects to decide and select the bio-signal for a defined motion.

The data was separated into batches for validation. Batch-1 comprised 60% data for training and 20% for validation and testing. Batch-2 trained on 70% of the data; 20% was separated for validation and 10% for testing. In other cases, the percentages for batch preparation varied. However, some batch sizes resulted in overfitting, while a couple was statistically insignificant when the student's t-test was performed. The training CA is 88.83 and 83.95% for sEMG and fNIRS, respectively. Each step was calculated in 230 μs with a 3 s epoch completion time. The training CA of 90.81 and 86.06% was observed when batch size was fixed at 600 samples for fNIRS and sEMG. Each step has been computed in 180 μs with a 2 s epoch completion time. When the batch size was explored in 400 for both fNIRS and sEMG, the CAs of 91.65 and 90.81% were achieved, respectively, while 130 μs has been observed with 2 s epoch completion time. An enhanced average of 94% predicted motions were found while evaluating the response from all healthy subjects for the fNIRS and sEMG activity alone, while the accuracy was 96% for four movements (Cui et al., 2010; Herold et al., 2017). This motion-wise accuracy is shown in Figure 7. This is where the modality of bio-signal was selected, and a defined motion was labeled to be predicted by the modality giving the best accuracy value. Paired *t*-test was implemented for sEMG vs. fNIRS vs. hybrid model accuracies, and by conventional criteria, this difference (*p* = 0.0001) is exceptionally statistically significant.

**Figure 8:**
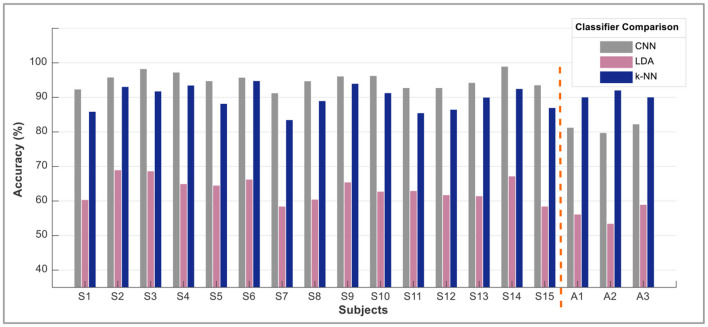
Subject-wise accuracies from each classifier.

**Figure 9:**
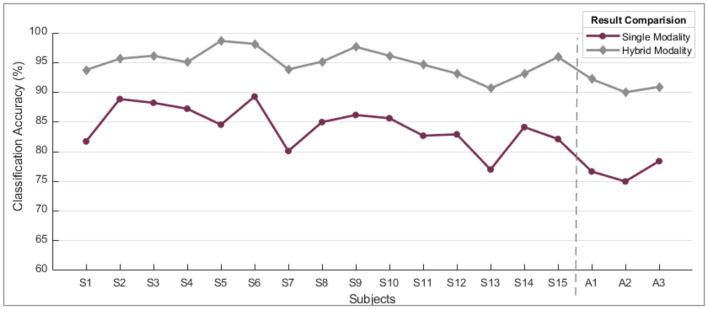
A comparison of single modality accuracies along with hybrid modality. These are the average values of accuracy computed for all motions subject-wise.

However, the sEMG classification accuracy for wrist flexion/extension was below the acceptable range of 80%. In the hybridization approach, weighted input activity produced 98% correct estimation by integrating fNIRS and surface electromyography. Subject-wise accuracies are given in Figure 8. The LDA and k-NN accuracies are extracted from the published studies that match the same sample set (Sattar et al., 2021b, 2022; Syed et al., 2021). Interestingly, the sEMG intentions for the wrist flexion/extension were weak, and hence fNIRS played a significant role in the inconsistency of the accuracy. The accuracy for the motion hybridized was increased to 98% as the fNIRS took command. It was also observed that the amputee A2 had amputation for over 10 years, which resulted in weak sEMG intentions. However, integrating the fNIRS and sEMG modalities helped achieve better accuracies. A graphical comparison of average classification accuracy evaluated from single modality signals and hybrid techniques is illustrated in Figure 9.

When comparing the performance of single modality vs. hybrid modality, it's clear that the combined approach consistently outperforms the gradual decline in accuracy observed with single modality. When the eminence of the myographic input dropped under a particular threshold value, the acquired outcomes were of poor quality. The solid infringement of the supposition that input patterns are stationary over time was the leading cause of failure. This problem was nullified by progressively updating the probabilities of the two input sources.

## 5. Control scheme for prosthetic arm device

**Figure 10:**
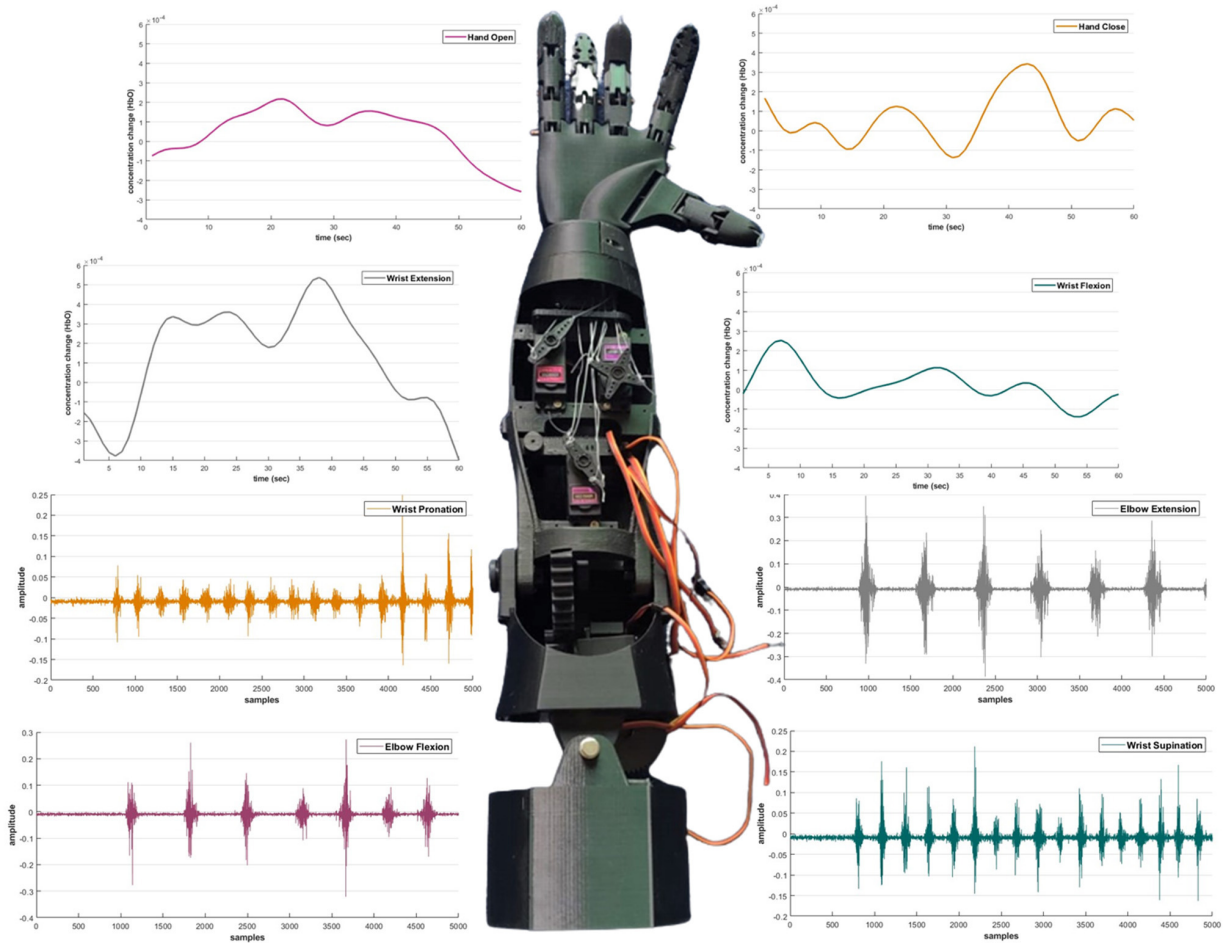
The prosthetic device control scheme based on electromyography and functional near-infrared spectroscopy signals.

The trained classifier was then implemented in real time to examine the potential of the proposed control scheme. The predicted motions were translated to actuate the prosthetic arm device, as shown in Figure 10. The control commands from both modalities was used to actuate the device according to the defined motion. As illustrated in Figure 10, each signal modality translated the four movements, resulting in the actuation of the prosthetic arm device. The device was tested on healthy as well as amputee subjects.

Unexpectedly, the combined command prompts an average increase of 6% in classification accuracy compared to the single modality scheme. The sEMG intentions are usually flawless and give no error while dealing with the classification of the signals. It has been observed that electromyographic sense may not always produce the desired outcome. Specifically, it has been noted that weak intention signals resulting from wrist motion can negatively impact overall performance. The real-time motion classification results are illustrated in Table 4.

**Table 4:** Real-time motion classification results of the proposed hybrid framework.

**Input class**	**No. of misclassified motions/no. of motions performed**	
	**Healthy**	**Amputee**
EE	0/30	0/20
EF	0/30	0/20
WS	0/30	0/20
WP	1/30	0/20
WE	0/30	1/10
WF	1/30	1/10
HO	0/30	1/15
HC	0/30	0/15

**Figure 11:**
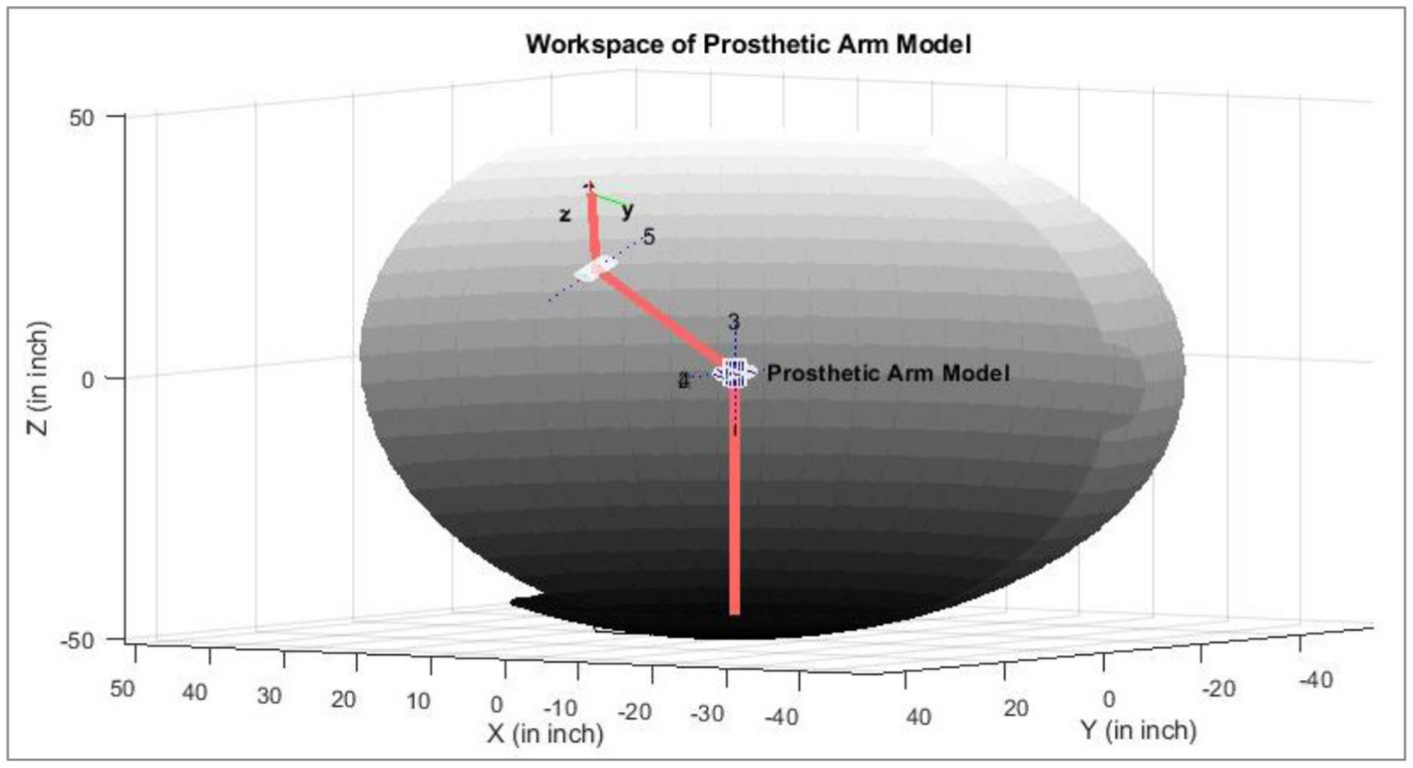
An example of trajectories of the prosthetic arm in the 3D space (healthy subject).

The workspace or the arm's trajectory varies as the arm length changes. The residual muscles of amputees also vary. Illustrated in Figure 11 is an example of the course that may be followed if the subject can perform the defined eight motions as discussed earlier. Determining the space/trajectory of the arm can be beneficial in future studies for robust real-time control.

To the authors' best knowledge, the proposed framework is unique in hybridizing sEMG and fNIRS signals for upper limb amputation. A comparison was made with other state-of-the-art machine learning techniques, such as LDA, SVM, k-NN, and ANN (Sattar et al., 2019, 2021a,b, 2022). The results are compared with the current research. The maximum individual classification accuracies and time taken by the machine learning algorithms are presented in Table 5.

**Table 5:** Performance evaluation and comparison with existing classification models (max. values).

**Technique**	**Learning method**	**Time response**			**Classification accuracy**		
		**sEMG**	**fNIRS**	**Hybrid**	**sEMG**	**fNIRS**	**Hybrid**
TD features	LDA	250 ms	1.5 s	2 s	65%	62.82%	67.12%
FD features	LDA/SVM	250 ms	2 s	2.5 s	N/A	N/A	52.67%
TFD features	k-NN	150 ms	1.5 s	1.5 s	73.70%	69.83%	74.5%
Raw sEMG	ANN	300 ms	N/A	N/A	72.30%	N/A	72.3%
Raw fNIRS	ANN	N/A	4 s	N/A	N/A	58%	58%
Raw sEMG/fNIRS	CNN	180 μs	230 μs	280 μs	86.83%	83%	88.83%
TD features	k-NN	150 ms	0.5s	0.5s	88.83%	86%	93.06%
**Proposed framework**	**CNN**	**130** **μs**	**200** **μs**	**180** **μs**	**91.81%**	**93.65%**	**98%**

Bold indicates differentiate between this study and previous works.

The present study validates the advantages of a hybrid neural-machine interface (Mughal et al., 2022). The hybridization of two distinct modalities resulted in stable output performance, unlike the results reported in published studies for a single modality source. Besides the exceptionally high adequacy, the intention from the growing muscular fatigue leads to a minor performance deprivation as the control is shifted to brain hemodynamic responses. Such a framework can be accounted for reliable hybrid brain-computer interface control for an extended day (Zafar et al., 2019).

Hybrid HMI techniques have the potential to provide a more intuitive and natural way for amputees to control their prostheses, allowing them to perform a broader range of tasks and activities. Some of the critical areas of research in this field include:

- Developing new algorithms and machine learning techniques to process fNIRS and sEMG signals and translate them into control commands for the prosthetic arm.
- Improvement of the accuracy and reliability of fNIRS and sEMG-based control systems by developing better sensors and signal processing methods.
- Hybrid fNIRS-sEMG control systems that can take advantage of the complementary information provided by these two modalities.

In general, the prospects for controlling prosthetic arms through the use of fNIRS and EMG signals are promising. There is continuous research being conducted to enhance and advance these technologies, which will undoubtedly play a vital role in the development of advanced prosthetic devices in the future.

## 6. Conclusion

The sEMG and fNIRS signals were acquired using the Thalamic Lab's Myo armband and a NIRSport from the NIRx Technology. The framework developed for real-time interfacing was investigated for eight-arm motion intention by humans. It includes WS, WP, WE, WF, EE, EF, HO, and HC. The bio-signals were analyzed for patterns related to the selected motions. A CNN was designed and implemented for the classification of the acquired bio-signals. The respective motion commands were generated for a prosthetic arm. The activity predicted by the proposed framework shows an accuracy of 94.5%.

Moreover, the highest value of accuracy of an individual subject was recorded at 98%. Such accuracy with eight control commands has yet to be reported. The time taken for the classifier to generate control commands and the accuracy were also evaluated through the state-of-the-art machine learning algorithms, and the results are compared with the proposed framework. The proposed scheme is a preprocessing step for making input evidence for future research. Future work will focus on the real-time implementation of the proposed system to control a prosthetic arm by the amputee. Furthermore, a framework to fuse these two bio-signals for fast command generation will be designed.

## Data availability statement

The raw data supporting the conclusions of this article will be made available by the authors, without undue reservation.

## Ethics statement

The studies involving human participants were reviewed and approved by Air University Human Research Ethics Committee (HREC). The patients/participants provided their written informed consent to participate in this study. Written informed consent was obtained from the individual(s) for the publication of any potentially identifiable images or data included in this article.

## Author contributions

All authors listed have made a substantial, direct, and intellectual contribution to the work and approved it for publication.
